# Phenolic Content, Antioxidant Capacity and In Vitro Glycemic Index of Traditional Noodle (Erişte) High in Plant‐Based Protein and *β*‐Glucan Content

**DOI:** 10.1002/fsn3.70481

**Published:** 2025-06-20

**Authors:** Damla Arer, Oguz Acar, Kubra Ozkan, Osman Sagdic, Andrea Visioni, Francesco Sestili, Hamit Koksel

**Affiliations:** ^1^ Department of Nutrition and Dietetics, Health Sciences Faculty Istinye University İstanbul Türkiye; ^2^ Department of Quality and Technology Field Crops Central Research Institute Ankara Türkiye; ^3^ Department of Food Engineering, Faculty of Chemical and Metallurgical Engineering Yildiz Technical University Istanbul Türkiye; ^4^ International Center for Agricultural Research in the Dry Areas (ICARDA) Rabat Morocco; ^5^ Department of Agriculture and Forest Sciences (DAFNE) University of Tuscia Viterbo Italy

**Keywords:** *β*‐glucan, erişte, in vitro glycemic index, plant‐based protein, traditional noodle

## Abstract

Traditional noodle samples (erişte) were supplemented with hull‐less barley and lentil flours as the source of *β*‐glucan and protein at different ratios and their cooking quality, phenolic content, antioxidant capacity and estimated GI values were evaluated. The estimated GI of control erişte produced from wheat flour was the highest (74.7), while GI of those supplemented with 15%, 30%, 45% barley or lentil flour were 68.7%, 66.0%, 61.2% and 67.5%, 63.8%, 60.6%, respectively. GI values of mixtures of barley and lentils flours (Mix‐1–4 samples) were lower (58.9–61.0). All noodles supplemented with barley and/or lentil flours had medium GI values. The erişte samples supplemented with 45% hull‐less barley flour and Mix erişte samples meet the requirements of FDA health claim (0.75 g *β*‐glucan per serving). Protein content of control sample was 16.30%, while those supplemented with lentil flour had higher protein contents (18.15%–22.36%). Hence, noodle samples supplemented with 30% and 45% lentil flour can be labeled as “high protein” and all other noodle samples can be labeled as “source of protein” according to EC Regulation because calories which can be received from proteins per serving are > 20% and > 12%, respectively. Significant increases were also observed in phenolic contents and antioxidant capacities of erişte samples supplemented with barley/lentil flours.

## Graphical abstract

Supplementation of erişte with 15%–45% hull‐less barley or lentil flour increased phenolic content, antioxidant capacity, *β*‐glucan or protein levels, and lowered glycemic index (GI), shifting all samples from high‐ to medium‐GI. Samples with 45% barley flour met FDA *β*‐glucan health claims. Those with 30% and 45% lentil flour qualify as “high in protein,” while others qualify as “source of protein” under Regulation (EC) 1924/2006. These findings highlight potential of barley and lentils in enhancing functional properties of traditional foods.

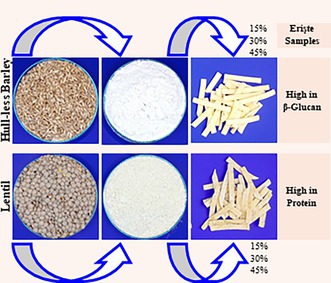

## 1 Introduction

The rise in metabolic diseases in the modern world has led to a recent growth in the food industry's manufacture of functional foods (Domínguez Díaz et al. 2020). Besides the food industry, consumer interest and demand for functional foods have also been increasing during the past few decades. Functional foods, rich in vitamins, amino acids, fatty acids, fiber, antioxidants, minerals, and probiotics, are believed to reduce oxidative stress, boost immunity and promote health (Abdulrazak and Jameel 2022). Traditional foods have great potential to serve as functional food if their nutritional compositions are improved by using suitable raw materials. However, there is still a lack of information on traditional foods, and further research is needed to recuperate their nutritional composition.

Erişte is a traditional noodle common in Central and western Asian countries, made from flour, water, salt, and occasionally egg or milk. The dough is sheeted, cut, and dried in the sun or oven. It is known as “erişte” in Turkish, “reshteh” in Persian, and “kesme” in Kazakh and various Central Asian countries (Shelke 2016). Although typically rich in carbohydrates, it is low in protein, fiber, and antioxidants. Its nutritional profile can be improved by using different cereals or functional ingredients (Bilgiçli 2009).

Plant proteins, especially from pulses and their isolates, are increasingly used as meat alternatives to reduce animal protein consumption (Magrini et al. 2018). This aligns with the UN Sustainable Development Goals. Pulses are widely used in foods for their high protein and functional properties like water/oil absorption, viscosity, foaming, and emulsification properties (Schmidt and Oliveira 2023). Their proteins also offer good nutritional quality due to relatively higher content of essential amino acids (Semba et al. 2021). Pulses are rich in several phenolic compounds, including flavonoids, which provide glycemic homeostasis through antioxidant activity and delayed glucose response and lipid digestion. The European Parliament and of the Council on Nutrition and Health Claims on foods have established a Regulation (EC) No 1924/2006. According to this regulation a food can be labeled as “source of protein” or “high in protein” where the protein content provides at least 12% or 20% of the energy value of the food, respectively. Pulses, like lentils, have strong potential as a sustainable protein source. Lentil flour is a protein‐rich, plant‐based alternative to wheat flour, offering high fiber, antioxidant activity, phytochemicals, and minerals, while being low in fat (Paucean et al. 2018).

Barley ( *Hordeum vulgare* L.) is the fourth important cereal after corn, rice, and wheat in the world in terms of production and cultivation area (Arendt and Zannini 2013). Although only about 6% of barley is used for human consumption, interest in its use for cereal‐based foods is growing (Tricase et al. 2018). Barley is rich in protein, starch, and dietary fiber, which is generally higher than in other cereals and varies by environment and genotype (Yalçın et al. 2007). It is considered a functional grain due to its *β*‐glucan and phytochemicals (FAO 2012). *β*‐glucans, key fibers in barley endosperm, are linked to lowering cholesterol, postprandial glucose, and insulin levels, while supporting the immune system (Sofi et al. 2019; Izydorczyk and McMillan 2019). Their viscosity and solubility influence intestinal function by delaying gastric emptying and glucose absorption (Zaremba et al. 2018).

The purpose of the present study is to obtain enriched erişte samples by using specific formulations including lentil flour as a source of protein and barley flour as a source of *β*‐glucan at different ratios and to evaluate the functional and textural characteristics as well as the estimated glycemic index (GI) values of the samples.

## 2 Materials and Methods

### 2.1 Material

In this study, a bread wheat cultivar ( *Triticum aestivum* L.), a hull‐less barley cultivar ( *Hordeum vulgare* L.) and an advanced lentil line were used for erişte production. The bread wheat cv. Tosunbey, a hard white wheat with strong gluten properties, was produced in the 2022–2023 season in Ankara (Türkiye) and was obtained from the Field Crops Central Research Institute (TARM). It was selected to compensate for the deteriorative effects of barley and lentil flour addition on gluten quality. The hull‐less barley cv. Chifaa, preferred for its high *β*‐glucan content, was produced during the 2022–2023 growing season in Marchouch (Morocco) and was obtained from the National Institute of Agricultural Research (INRA). The advanced lentil line with high protein content was produced in Lebanon and was obtained from the International Center for Agricultural Research in the Dry Areas (ICARDA). Salt and pasteurized eggs were purchased from local markets in Türkiye. The solvents and reagents were all obtained from Sigma‐Aldrich (St. Louis, MO, USA). The assay kits for *β*‐glucan and glucose were purchased from Megazyme International (Wicklow, Ireland).

### 2.2 Methods

#### 2.2.1 Milling of Samples

Refined flour of wheat and hull‐less barley samples were obtained by milling according to Approved Method 26‐50 (AACC International 2010) by using a Buhler MLU 202 Pneumatic Laboratory Mill (Uzwil, Sweden). The lentil sample was ground by using a laboratory type mill (Perten LM 3100 Laboratory Mill, Huddinge, Sweden).

#### 2.2.2 Production of Erişte Samples

Hull‐less barley and lentil flour were added to the wheat flour at ratios of 15%, 30%, and 45%. Hull‐less barley and lentil flour were also added to wheat flour together and designated as Mix erişte samples. The ratios of wheat flour, hull‐less barley, and lentil flour in Mix‐1, Mix‐2, Mix‐3, and Mix‐4 were 40%, 45%, 15%; 35%, 50%, 15%; 40%, 50%, 10%; and 35%, 50%, 15% respectively.

Erişte samples were produced according to the modified method of Demir et al. (2021) using 200 g flour (14% moisture basis) 3 g salt, 40 g pasteurized eggs, and water at 30°C. The amount of water was determined by preliminary tests based on the Farinograph water absorption values. First, the dry ingredients, wheat flour, barley flour, and salt were mixed with a spatula. Then it was mixed with a dough mixer (Kitchen Aid, Classic 4.3 L, St. Joseph, MI, USA) for 1 min to prepare a homogeneous dry mixture. Then, pasteurized egg and water as liquid ingredients were added and mixed for 6 min. The dough surface was covered to prevent drying and left to rest for 20 min. The rested dough was pre‐sheeted twice with a rolling pin, once on the front and once on the back side. The dough was then sheeted twice using a dough sheeting equipment (Hamak Makina, Türkiye). The dough sheets were left to partially rest and decrease surface stickiness for 1 h at room temperature (25°C, 31% humidity). After that, dough samples were further sheeted by passing the dough sheets through the cylinders of the sheeting equipment once (Hamak Makina, Türkiye) adjusted with a gap of 5.4 mm.

#### 2.2.3 Determination of Various Chemical Characteristics

The moisture, protein, ash, and *β*‐glucan contents were determined according to the Approved Methods 44‐15.02, 46‐30.01, 08‐01.01, and 32‐23.01, respectively (AACC International 2010).

#### 2.2.4 Determination of Cooking, Textural and Color Properties

Cooking properties (cooking time, water absorption, volume increase and cooking loss) were determined on 20 g of erişte sample. The cooking time of erişte samples determined according to method of Basman and Yalcin (2011). The water absorption, volume increase, and cooking loss of the erişte samples were determined according to the formulas below.

$$\text{Water absorpsion}\;\left(\%\right)=\frac{\text{Weight of cooked erişte}\;\left(g\right)-\text{weight of uncooked erişte}\;\left(g\right)\;}{\text{Weight of uncoooked erişte}\;\left(g\right)}\times 100$$

$$\text{Volume increase}\;\left(\%\right)=\frac{\text{Volume of cooked erişte}\;\left(\mathrm{mL}\right)-\text{Volume of uncooked erişte}\;\left(\mathrm{mL}\right)}{\text{Volume of uncoooked erişte}\;\left(\mathrm{mL}\right)}\times 100$$

$$\text{Cooking Loss}\;\left(\%\right)=\frac{\text{The weight of the residue in the baker}\;}{\text{The weight of the starting erişte material}}\times 100$$

Textural properties of erişte samples were determined by using a texture analyzer (Stable Microsystems, TA‐XT plus, Godalming, Surrey, England) according to Approved Method 16‐50 (AACC International 2010). The color values (L*, a* and b*) of samples were measured using a colorimeter (BYK Gardner Color‐View, USA). Total color difference (∆*E*) was computed using the following formula:

$$\Delta E=\sqrt{{\left(L^{*}-L_{0}^{*}\right)}^{2}+{\left(a^{*}-a_{0}^{*}\right)}^{2}+{\left(b^{*}-b_{0}^{*}\right)}^{2}}$$

#### 2.2.5 Determination of In Vitro Glycemic Index Value

Glucose Assay Kit (Megazyme Int., Wicklow, Ireland) was used for the determination of in vitro GI values of erişte samples after cooking following the protocols established by Aribas et al. (2020). The glucose content was measured using a spectrophotometer (Shimadzu 150 UV‐1800 spectrophotometer, Kyoto, Japan) set to 510 nm wavelength and glucose oxidase–peroxidase (GOPOD) reagent (Megazyme Int., Ireland).

#### 2.2.6 Determination of Phenolic Compounds and Antioxidant Capacities

Free and bound phenolic compounds of the ground erişte samples were extracted according to Shamanin et al. (2022). To determine the concentrations of free and bound phenolic compounds, a modified Folin–Ciocalteu method was used (Singleton and Rossi 1965).

The antioxidant capacity was determined as described by Singh et al. (2002) with modifications. 100 μL of the erişte extract was mixed with 4.9 mL of the DPPH solution after 60 min of incubation at 27°C. The ABTS scavenging capacity was determined according to Re et al. (1999). The FRAP method developed by Benzie and Strain (1996) was used with minor modifications. The absorbance values were measured at 517, 734, and 593 nm, respectively (Shimadzu 150 UV‐1800 spectrophotometer, Kyoto, Japan).

#### 2.2.7 Statistical Analysis

The results were expressed as the means of at least duplicate measurements. Analysis of variance (ANOVA) was performed to determine significant differences. When significant differences (*p* < 0.01 or *p* < 0.05) were found, the least significant difference (LSD) method was applied to compare the means. Furthermore, correlation coefficients were calculated between various parameters. All statistical analysis was performed with software JMP (version 13.2.1, SAS Institute Inc 2013).

## 3 Results and Discussion

### 3.1 Impact of Barley and Lentil Flour Supplementation on the Chemical, Cooking, Textural, and Color Properties of Erişte

Various chemical properties of the flour samples used in erişte production are shown in Table 1. The *β*‐glucan content of the flour obtained from hull‐less barley was 3.02% while the protein content of the flour obtained from lentil was 29.76%. As indicated, the hull‐less barley cultivar Chifaa and the advanced lentil line were selected to enhance *β*‐glucan and protein contents of erişte samples, respectively. In a previous study, hull‐less barley was used in the form of whole grain flour without separation of bran, which resulted in deterioration in the textural properties of flatbread, especially the hardness and chewiness values (Koksel et al. 2024). Therefore, in the present study, refined hull‐less flour was used after separation of bran. However, it seems that *β*‐glucans were also removed together with bran during the milling process since the *β*‐glucan contents of barley grain and its refined flour were 6.62% and 3.02% (db), respectively.

**TABLE 1:** Various chemical properties of flours used in erişte production.

Sample	Moisture (%)	Ash (%, db)	Protein (%, db)	*β*‐glucan (%, db)
Wheat flour	13.43 ± 0.04	0.47 ± 0.09	14.33 ± 0.00	0.40 ± 0.02
Barley flour	10.77 ± 0.03	1.41 ± 0.03	12.54 ± 0.01	3.02 ± 0.01
Lentil	9.26 ± 0.01	2.98 ± 0.02	29.76 ± 0.14	0.06 ± 0.01

Abbreviation: db, dry basis.

**FIGURE 1:**
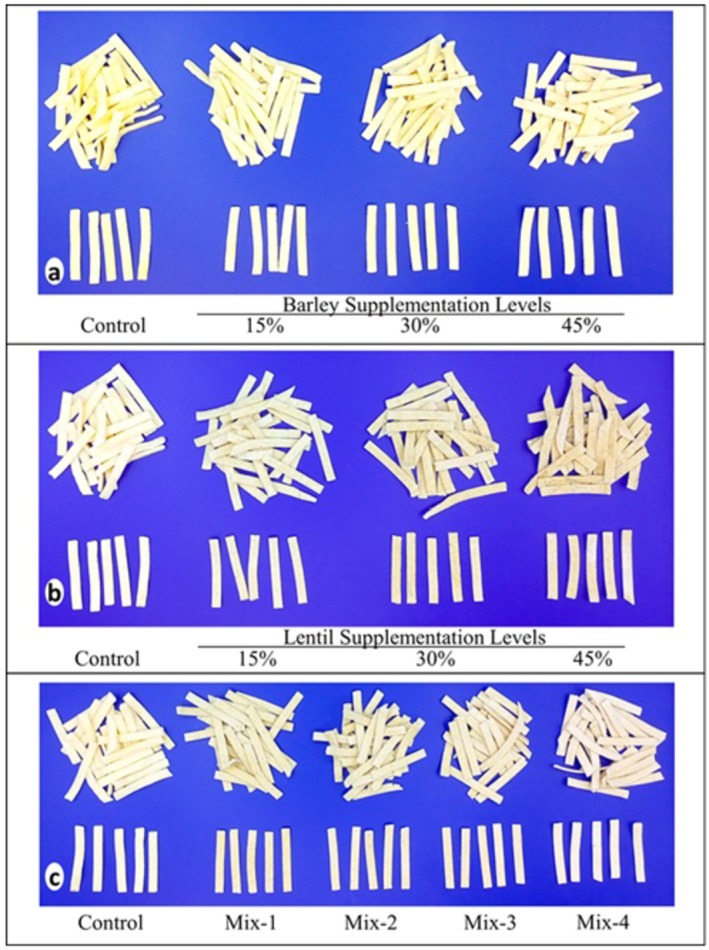
Supplemented with hull‐less barley (a), lentil (b), and mix (c) erişte samples.

The images in Figure 1 are provided for visual comparison of erişte samples while their cooking and textural properties and color values are given in Table 2. All of the erişte samples supplemented with barley or lentils had significantly higher cooking time (except the one supplemented with 15% lentils), water absorption (except the ones supplemented with barley flour), volume increase (except the ones supplemented with lentils) and cooking loss (except the ones supplemented with barley flour) as compared to the control sample produced by using wheat flour (*p* < 0.05). Glutenins and gliadins, the two insoluble proteins that constitute wheat gluten, form intra‐ and inter‐molecular disulfide bonds during dough processing, creating a three‐dimensional gluten network capable of entrapping starch granules within the gluten matrix (Ma et al. 2022). In contrast pulse proteins consist of mainly soluble proteins (globulin and albumin). Hence, the inclusion of non‐gluten proteins, such as those found in pulse flours, weakens the gluten network and reduces its ability to retain starch granules in gluten matrix during cooking. This likely explains the higher cooking loss observed in erişte samples supplemented with lentil flour.

**TABLE 2:** Cooking, color, and textural properties of erişte samples.

Sample	Cooking time (min)	Water absorption (%)	Volume increase (%)	Cooking loss (%)	L*	a*	b*	ΔE*	Firmness (g)	Work of shear (g · cm)
Control	14.5 ± 0.50 c	138.1 ± 3.57 b	153.5 ± 9.03 c	5.3 ± 0.20 a	73.8 ± 0.55 a	7.0 ± 0.19 a	31.5 ± 0.41 a	—	451.2 ± 35.70 a	76.4 ± 8.53 a
15% Barley	15.8 ± 0.25 b	164.7 ± 9.61 a	197.1 ± 2.94 a	5.0 ± 0.23 a	72.1 ± 0.45 b	6.3 ± 0.21 b	28.2 ± 0.46 b	3.8 ± 0.61 b	477.8 ± 53.13 a	76.2 ± 10.21 ab
30% Barley	16.2 ± 0.15 b	166.7 ± 5.62 a	194.2 ± 5.88 a	5.6 ± 0.05 a	70.5 ± 0.19 c	5.8 ± 0.09 c	26.4 ± 0.17 c	6.2 ± 0.12 a	422.7 ± 30.64 a	62.0 ± 7.34 bc
45% Barley	17.5 ± 0.00 a	161.0 ± 7.81 a	184.6 ± 9.56 a	6.0 ± 0.19 a	71.1 ± 0.22 c	5.5 ± 0.21 d	25.7 ± 0.33 d	6.6 ± 0.38 a	411.8 ± 33.71 a	58.1 ± 8.82 c
Control	14.5 ± 0.50 c	138.13 ± 3.57 b	153.5 ± 9.03 a	5.3 ± 0.20 c	73.8 ± 0.55 a	7.0 ± 0.19 a	31.5 ± 0.41 a	—	451.2 ± 35.70 c	76.4 ± 8.53 a
15% Lentil	15.8 ± 0.25 bc	158.0 ± 4.82 a	194.5 ± 18.01 a	5.8 ± 0.26 bc	66.3 ± 1.01 b	4.6 ± 0.41 b	29.2 ± 0.76 b	8.2 ± 1.15 c	502.9 ± 49.45 bc	75.2 ± 5.51 a
30% Lentil	17.5 ± 0.50 a	162.1 ± 1.05 a	173.6 ± 2.94 a	6.5 ± 0.01 ab	62.7 ± 0.89 c	4.9 ± 0.18 b	29.4 ± 0.24 b	11.6 ± 0.86b	576.4 ± 34.32 a	85.4 ± 8.60 a
45% Lentil	17.3 ± 0.25 ab	163.1 ± 2.85 a	191.2 ± 8.82 a	6.9 ± 0.27 a	61.2 ± 0.52 d	4.7 ± 0.07 b	28.5 ± 0.64 b	13.1 ± 0.54 a	574.5 ± 42.07 ab	77.9 ± 6.75 a
Control	14.5 ± 0.50 d	138.1 ± 3.57 c	153.5 ± 9.03 c	5.3 ± 0.20 b	73.8 ± 0.55 a	7.0 ± 0.19 a	31.5 ± 0.41 a	—	451.2 ± 4.01 b	76.4 ± 8.53 a
Mix‐1	18.5 ± 0.00 ab	163.9 ± 4.83 ab	223.4 ± 3.33 a	7.0 ± 0.40 a	60.2 ± 0.21 d	4.8 ± 0.13 c	24.7 ± 0.29bc	15.4 ± 0.05 a	515.1 ± 31.88 a	64.5 ± 1.91 b
Mix‐2	19.0 ± 0.00 a	169.4 ± 1.27 a	206.9 ± 13.13 ab	7.0 ± 0.19 a	61.1 ± 034 c	4.6 ± 0.06 c	24.0 ± 0.51 c	14.9 ± 0.28 ab	486.4 ± 28.36 ab	59.4 ± 4.37 b
Mix‐3	17.5 ± 0.50 bc	156.0 ± 0.35 b	184.4 ± 3.13 b	7.1 ± 0.47 a	60.7 ± 0.35 cd	5.1 ± 0.16 b	24.9 ± 0.32 b	14.9 ± 0.17 b	526.6 ± 28.36 a	63.2 ± 4.03 b
Mix‐4	17.3 ± 0.00 c	160.0 ± 3.08 ab	206.7 ± 6.67 ab	7.0 ± 0.31 a	62.5 ± 0.12 b	4.9 ± 0.09 bc	23.9 ± 0.39 c	13.8 ± 0.30 c	510.8 ± 14.61 a	64.7 ± 3.77 b

*Note:* Means followed by different letters in columns are significantly different at *p* < 0.05. Each group (barley or lentils supplemented or Mix samples) was analyzed and compared separately.

In the present study, the firmness values of erişte samples supplemented with lentils were significantly higher than that of the control and increased with a higher level of lentil supplementation (*p* < 0.05). In a study in which lentil flour was used to enrich pasta samples, the firmness values increased in line with the present study (Di Stefano et al. 2020). Lentil flour supplementation had no significant effect on the work of shear values of erişte samples. On the other hand, the enrichment process resulted in a significant decrease in L*, a*, and b* color values (*p* < 0.05). This means the erişte samples supplemented with barley or lentil flour were less bright and less yellow as compared to the control sample. A significant increase was also observed in ΔE* values with the increasing levels of barley and lentils. However, in a study conducted by Koksel et al. (2024) the b* values of the flatbread samples supplemented with barley flour were higher than those of the control sample in terms of both crust and crumb color. The contrary results in these studies might have been caused by the differences in production methods of flatbread and noodle samples. Furthermore, in that study, hull‐less barley flour was used in the form of whole grain flour without separation of bran.

### 3.2 Nutritional Composition, *β*‐Glucan Content, and Glycemic Index of Erişte Supplemented With Barley and Lentil Flours

The protein and *β*‐glucan content along with the estimated in vitro GI values of control and erişte samples supplemented with different levels of barley and lentil flours are presented in Table 3. Significant differences were observed among the erişte samples for these parameters (*p* < 0.05). The protein contents of the erişte samples supplemented with lentil flour were determined as 18.15%, 20.56%, and 22.36% while those of erişte samples supplemented with barley flour were 16.19%, 15.85%, and 15.29% with the addition of 15%, 30%, and 45% lentil or barley flour, respectively. In addition, the protein contents of Mix samples were determined in the range between 16.62% and 18.23%. Lentils are relatively lower in carbohydrates and higher in protein compared to cereals. Hence, supplementation of higher amounts of lentil flour resulted in significantly higher protein levels in erişte samples (*p* < 0.05). According to Regulation (EC No 1924/2006) by the European Parliament and the Council on Nutrition and Health Claims on Foods, there are two classifications for protein related nutrition claims, “source of protein” and “high in protein” on the label of a food that contains protein. This claim states that a product can be classified as a “source of protein” if its protein content accounts for at least 12% of its energy value and as “high in protein” if its protein content accounts for at least 20% of its energy value. In the present study, the conversion factors used for the erişte samples were 4, 4, and 9 kcal/g for carbohydrate, protein, and fat, respectively, in accordance with the Guidelines on Nutrition Labeling (Codex Alimentarius Commission 1985). By using these factors, the calories which can be received from proteins of the erişte samples supplemented with barley flour or lentil flour and mix samples were determined in the range of 15.1%–16.0%, 17.9%–22.1%, and 16.4%–18.0%, respectively. Based on to these results, the erişte samples enriched with 30% and 45% lentil flour can be labeled as “high in protein” according to the Regulation (EC) No 1924/2006 as the proportion of calories derived from protein in these samples was 20.3% and 22.1%, respectively. On the other hand, the other erişte samples can be labeled as “source of protein” according to the same regulation since the calories from proteins of these erişte samples is higher than 12% but lower than 20%.

**TABLE 3:** Protein and *β*‐glucan contents and estimated in vitro glycemic index properties of erişte samples.

Sample	Protein content (%, db)	*β*‐glucan content (%, db)	In vitro glycemic index
Control	16.30 ± 0.007 a	0.38 ± 0.013 d	74.7 ± 0.084 a
15% Barley	16.19 ± 0.137 a	0.85 ± 0.018 c	68.7 ± 0.506 b
30% Barley	15.85 ± 0.03 b	1.00 ± 0.004 b	66.0 ± 0.285 c
45% Barley	15.29 ± 0.074 c	1.62 ± 0.004 a	61.2 ± 0.267 d
Control	16.30 ± 0.007 d	0.38 ± 0.013 a	74.7 ± 0.084 a
15% Lentil	18.15 ± 0.236 c	0.36 ± 0.004 a	67.5 ± 0.244 b
30% Lentil	20.56 ± 0.213 b	0.24 ± 0.018 b	63.8 ± 0.351 c
45% Lentil	22.36 ± 0.177 a	0.18 ± 0.004 c	60.6 ± 0.372 d
Control	16.30 ± 0.007 d	0.38 ± 0.013 e	74.7 ± 0.084 a
Mix‐1	18.23 ± 0.112 a	1.16 ± 0.018 d	61.0 ± 0.086 b
Mix‐2	17.96 ± 0.112 a	1.35 ± 0.003c	58.9 ± 0.143 d
Mix‐3	17.09 ± 0.056 b	1.53 ± 0.014 b	59.7 ± 0.263 c
Mix‐4	16.62 ± 0.057 c	1.59 ± 0.010 a	60.7 ± 0.084 b

*Note:* Means followed by different letters in columns are significantly different at *p* < 0.05. Each group (barley or lentils supplemented or Mix samples) was analyzed and compared separately.

Abbreviation: db, dry basis.

The *β*‐glucan contents of erişte samples supplemented with 15%, 30%, and 45% barley flour were determined as 0.85%, 1.00%, and 1.62%, respectively. As the level of barley flour supplementation increased, the *β*‐glucan content also increased, while it decreased with lentil flour supplementation. The *β*‐glucan contents of Mix‐1, Mix‐2, Mix‐3, and Mix‐4 erişte samples supplemented with various proportions of wheat, barley, and lentil flours ranged from 1.16% to 1.59%. According to the Food and Drug Administration (FDA), an intake of 3 g of *β*‐glucan soluble fiber per day is required to lower serum cholesterol levels and reduce the risk of coronary heart disease. To qualify for the health claim, foods must provide at least 0.75 g of *β*‐glucan soluble fiber/serving divided over four eating occasions (three meals and a snack) (FDA 2005). The standard serving size of dried pasta is reported as 80 g (Dello Russo et al. 2021). Based on the results of the present study, it is estimated that consuming 80 g of erişte samples supplemented with 45% hull‐less barley flour, Mix‐1, Mix‐2, Mix‐3, and Mix‐4 erişte samples could provide approximately 1.18, 0.83, 0.96, 1.09, and 1.11 g of *β*‐glucan, respectively.

Based on these results, the erişte samples enriched with 45% hull‐less barley flour and Mix erişte samples meet the requirements of the health claim (FDA 2005) by providing the necessary amount of *β*‐glucan per serving (0.75 g) with a consumption of 80 g erişte sample. In a study conducted by Ficco et al. (2023), barley flour was incorporated at different ratios (2%, 5%, 7% and 10%) to enrich and improve the nutritional quality of bread. The results demonstrated that the supplementation with barley flour increased the *β*‐glucan content of samples. Similarly, a study by Koksel et al. (2024) reported a significant increase in the *β*‐glucan content of flatbread samples supplemented with barley flour, which ranged from 0.79% to 2.83% on a dry basis for supplementation levels of 15% to 60%. According to these results, the breads supplemented with 45% and 60% barley flour meet the requirements to carry the health claim by providing the required amount of *β*‐glucan (3 g) per day with the consumption of 200–250 g of flatbread. These results align with the present study, where erişte samples supplemented with 45% barley flour, Mix‐3, and Mix‐4 also meet the criteria for the health claim by providing sufficient *β*‐glucan content.

According to GI values, foods can be classified as low (GI ≤ 55), medium (GI 56–69) and high (GI ≥ 70) GI foods (Kumar et al. 2018). The in vitro GI values of erişte samples decreased with increasing barley flour supplementation level and ranged from 61.17 to 68.61. The in vitro GI values of the erişte samples supplemented with 15%, 30% and 45% lentil flour were 67.47, 63.71 and 60.55, respectively, while the in vitro GI values of Mix‐1, Mix‐2, Mix‐3, and Mix‐4 erişte samples (containing different levels of barley flour, lentil flour and wheat flour) were 60.99, 58.90, 59.68 and 60.68, respectively. In contrast, the control erişte sample had a GI value of 74.61, classifying it as a high GI food (GI ≥ 70). Supplementation with barley and lentil flours significantly reduced the GI values, reclassifying these samples as medium GI foods. These findings align with a recent study (Koksel et al. 2024), which reported that the in vitro GI values of flatbread supplemented with 15%, 30%, 45% and 60% barley flour decreased as the level of barley flour increased.

### 3.3 Phenolic Content and Antioxidant Capacity of Erişte Enriched With Barley and Lentil Flour

Phenolic compounds and antioxidant capacities of the control erişte and those supplemented with barley flour are presented in Table 4. In erişte samples supplemented with barley flour (15%, 30% and 45%), both free and bound phenolic contents increased with increasing level of barley flour. Free phenolic contents (FPC) of the control and erişte samples supplemented with barley flour ranged from 199.84 to 265.49 mg GAE/100 g (db), while their bound phenolic contents (BPC) were in the range of 207.32 to 267.22 mg GAE/100 g (db). Among these, the erişte sample supplemented with 45% barley flour had the highest content of bound phenolic compounds (267.22 mg GAE/100 g, db). As expected, the bound phenolic contents of the erişte samples supplemented with barley flour were higher than the free form. Bound phenolics are covalently linked to the structural components of the cereal cell walls and are mostly found in bound form in matrices (Idehen et al. 2017). Total phenolic contents (TPC) of the control and erişte samples supplemented with barley flour ranged from 407.16 to 532.70 mg GAE/100 g (db) and increased with increasing supplementation level. In a study on bread formulation, it was reported that substituting wheat flour with barley flour enhanced the amount of total phenolic compounds and antioxidant activity. The substitution with 60% barley flour increased the total phenolics content and the antioxidant activity of loaves around 42% and 45%, respectively (del Carmen Robles‐Ramírez et al. 2020).

**TABLE 4:** Phenolic compounds and antioxidant capacities of the erişte samples supplemented with hull‐less barley in different ratios.

	Sample	Phenolic content (mg GAE/100 g, db)	DPPH (mg TE/100 g, db)	FRAP (mg TE/100 g, db)	ABTS (mg TE/100 g, db)
Free	Control	199.84 ± 1.244 d	41.44 ± 1.395 d	5.83 ± 0.642 d	46.76 ± 1.008 d
	15% Barley	212.32 ± 0.990 c	46.70 ± 0.856 c	10.45 ± 0.728 c	50.57 ± 0.635 c
	30% Barley	218.46 ± 1.183 b	59.77 ± 0.846 b	13.90 ± 0.642 b	70.58 ± 0.762 b
	45% Barley	265.49 ± 1.165 a	66.78 ± 0.554 a	24.88 ± 0.803 a	78.63 ± 1.143 a
Bound	Control	207.32 ± 1.244 d	50.95 ± 1.395 c	52.08 ± 0.951 c	61.62 ± 0.519 d
	15% Barley	214.84 ± 0.823 c	68.66 ± 1.801 b	59.42 ± 0.555 b	76.10 ± 0.504 c
	30% Barley	220.57 ± 0.940 b	79.22 ± 1.154 a	61.10 ± 0.667 b	84.13 ± 0.660 b
	45% Barley	267.22 ± 2.544 a	79.22 ± 1.693 a	97.55 ± 1.890 a	89.12 ± 0.762 a
Total	Control	407.16 ± 0.814 d	92.39 ± 2.239 d	57.91 ± 1.391 d	108.38 ± 1.087 d
	15% Barley	427.16 ± 1.452 c	115.36 ± 1.172 c	69.86 ± 1.024 c	126.66 ± 1.019 c
	30% Barley	439.02 ± 2.120 b	138.99 ± 1.466 b	75.00 ± 0.626 b	154.71 ± 1.374 b
	45% Barley	532.70 ± 4.223 a	146.00 ± 1.946 a	122.43 ± 1.784 a	167.74 ± 1.804 a

*Note:* Means followed by different letters in columns are significantly different at *p* < 0.05. Each group (free, bound or total) was analyzed and compared separately.

Abbreviation: db, dry basis.

It is a common practice to evaluate the antioxidant activities of cereals and cereal products using different methods due to their complex structures. In the present study, the antioxidant capacities of erişte samples were determined by DPPH, ABTS, and FRAP methods (Table 4). The total ABTS values of the control and erişte samples supplemented with barley flour ranged from 108.38 to 167.74 mg TE/100 g (db). Their total DPPH and FRAP values were in the range of 92.39–146.00 and 57.91–122.43 mg TE/100 g (db), respectively. As the supplementation level of hull‐less barley flour in the erişte samples increased, the DPPH, ABTS, and FRAP values also increased compared to the control erişte sample. A study by Holtekjølen et al. (2008) demonstrated that the antioxidant capacity of bread enriched with 40% barley flour was higher than the control bread sample (100% wheat flour). Similarly, Nakov et al. (2022) reported that cookies with the addition of hulled barley flour had higher TPC and antioxidant capacity compared to control cookies (100% wheat flour). In another study conducted by Punia et al. (2020), increasing the proportion of barley flour in wheat‐barley flour mixtures of rusks with 10%, 20%, 30%, 40%, and 50% barley flour substitution to wheat flour gradually increased the antioxidant activity from 15% to 31%. This enrichment in antioxidant activity was attributed to the inherently higher antioxidant activity of barley flour compared to wheat flour.

Phenolic compounds and antioxidant capacities of the control erişte and those supplemented with lentil flour are presented in Table 5. The FPC of control and erişte samples supplemented with lentil flour ranged from 199.84 to 238.52 mg GAE/100 g (db), while the BPC of erişte samples varied between 207.32 and 239.88 mg GAE/100 g (db). As the lentil flour supplementation level of the erişte samples increased, the TPC also increased compared to the control erişte sample. The total DPPH values of erişte samples supplemented with lentil flour were in the range of 113.30–143.95 mg TE/100 g (db), with the control sample showing the lowest DPPH values of free, bound, and total forms. Similar to the erişte samples supplemented with hull‐less barley flour, the antioxidant capacities of erişte samples increased with higher levels of lentil flour supplementation when compared to the control sample. Cacak‐Pietrzak et al. (2024) reported that the TPC value of control bread made by using 100% wheat flour was 46 mg GAE/100 g (db), and the TPC value of breads supplemented with lentil flour at the ratios 10%, 20% and 25% were determined as 55, 61, 75 and 85 mg GAE/100 g (db), respectively. The same authors reported that breads supplemented with lentil flour had higher phenolic content than the control bread (100% wheat flour) and the phenolic content of the breads increased as the lentil flour addition level increased. Similarly, Portman et al. (2020) determined that FRAP values of cookies significantly increased with increasing level of lentil flour.

**TABLE 5:** Phenolic compounds and antioxidant capacities of the erişte samples supplemented with lentil flour in different ratios.

	Sample	Phenolic content (mg GAE/100 g, db)	DPPH (mg TE/100 g, db)	FRAP (mg TE/100 g, db)	ABTS (mg TE/100 g, db)
Free	Control	199.84 ± 1.244 d	41.44 ± 1.395 c	5.83 ± 0.642 d	46.76 ± 1.008 d
	15% Lentil	210.96 ± 1.196 c	48.30 ± 0.970 b	15.96 ± 0.632 c	53.34 ± 0.525 c
	30% Lentil	216.98 ± 1.452 b	67.51 ± 2.020 a	29.27 ± 0.312 b	62.30 ± 0.385 b
	45% Lentil	238.52 ± 1.196 a	70.72 ± 1.166 a	49.71 ± 0.550 a	74.12 ± 1.636 a
Bound	Control	207.32 ± 1.244 d	50.95 ± 1.395 c	52.08 ± 0.951 b	61.62 ± 0.219 d
	15% Lentil	212.32 ± 0.990 c	65.00 ± 1.410 b	38.76 ± 0.764 c	65.90 ± 0.504 c
	30% Lentil	217.17 ± 1.258 b	68.43 ± 1.712 ab	39.42 ± 0.662 c	68.99 ± 0.667 b
	45% Lentil	239.88 ± 0.951 a	73.23 ± 2.264 a	65.37 ± 0.127 a	92.57 ± 0.635 a
Total	Control	407.16 ± 0.814 d	92.39 ± 2.239 d	57.91 ± 1.391 c	108.38 ± 1.087 d
	15% Lentil	423.28 ± 2.178 c	113.30 ± 2.264 c	54.72 ± 0.375 d	119.25 ± 0.955 c
	30% Lentil	434.15 ± 2.703 b	135.94 ± 1.166 b	68.69 ± 0.624 b	131.29 ± 0.886 b
	45% Lentil	478.39 ± 1.196 a	143.95 ± 2.911 a	115.08 ± 0.579 a	166.68 ± 1.350 a

*Note:* Means followed by different letters in columns are significantly different at *p* < 0.05. Each group (free, bound or total) was analyzed and compared separately.

Abbreviation: db, dry basis.

The phenolic compounds and antioxidant capacities of the control and Mix erişte samples are presented in Table 6. The TPC of Mix‐1 and Mix‐2 erişte samples was 565.30 and 572.45 mg GAE/100 g (db), respectively. The ratios of barley flour in Mix‐2 and Mix‐3 erişte samples were the same (50%) and their TPC values were determined as 572.45 and 548.40 mg GAE/100 g (db), respectively. This difference is probably due to the higher level of lentil flour in Mix‐2. The FPC of Mix‐2 erişte sample was 284.08 mg GAE/100 g (db), while its BPC was 288.37 mg GAE/100 g (db). The Mix‐2 erişte sample had the highest free, bound, and total ABTS values, which were found as 111.59, 113.28, and 224.87 mg TE/100 g (db), respectively, while the Mix‐1 erişte sample had the lowest values in all categories. The correlation coefficients between the total phenolic content (TPC) and the antioxidant capacities of erişte samples, determined by DPPH, ABTS, and FRAP methods, were also calculated. The results revealed significant correlations between TPC and the antioxidant assays, with correlation coefficients of *r* = 0.933 for DPPH, *r* = 0.939 for ABTS, and *r* = 0.954 for FRAP (*p* < 0.001).

**TABLE 6:** Phenolic compounds and antioxidant capacities of the mix erişte samples.

	Sample	Phenolic content (mg GAE/100 g, db)	DPPH (mg TE/100 g, db)	FRAP (mg TE/100 g, db)	ABTS (mg TE/100 g, db)
Free	Control	199.84 ± 1.244 c	41.44 ± 1.395 c	5.83 ± 0.642 d	46.76 ± 1.008 d
	Mix‐1	281.94 ± 1.684 a	97.80 ± 0.573 a	40.75 ± 0.664 c	98.86 ± 0.788 c
	Mix‐2	284.08 ± 1.782 a	95.92 ± 1.442 a	43.24 ± 0.552 b	111.59 ± 1.031 a
	Mix‐3	272.89 ± 1.543 b	94.99 ± 1.146 a	49.63 ± 0.647 a	108.54 ± 0.788 ab
	Mix‐4	268.13 ± 0.891 b	84.47 ± 1.516 b	48.35 ± 0.592 a	106.22 ± 1.547 b
Bound	Control	207.32 ± 1.244 d	50.95 ± 1.395 d	52.08 ± 0.951 d	61.62 ± 0.519 d
	Mix‐1	283.37 ± 2.048 a	99.20 ± 1.146 b	82.25 ± 1.034 b	101.28 ± 0.649 c
	Mix‐2	288.37 ± 1.782 a	105.28 ± 1.442 a	87.22 ± 0.689 a	113.28 ± 0.394 a
	Mix‐3	275.51 ± 2.357 b	98.03 ± 1.442 b	77.30 ± 1.584 c	110.54 ± 0.537 b
	Mix‐4	267.65 ± 2.048 c	85.64 ± 0.875 c	76.01 ± 1.017 c	111.70 ± 1.073 ab
Total	Control	407.16 ± 0.814 e	92.39 ± 2.239 d	57.91 ± 1.391 d	108.38 ± 1.087 d
	Mix‐1	565.30 ± 0.891 b	196.99 ± 0.573 ab	123.01 ± 0.368 c	200.13 ± 0.773 c
	Mix‐2	572.45 ± 0.676 a	201.20 ± 1.46 a	130.46 ± 0.908 a	224.87 ± 0.649 a
	Mix‐3	548.40 ± 3.121 c	193.02 ± 1.442 b	126.92 ± 0.647 b	219.08 ± 1.124 b
	Mix‐4	535.78 ± 2.916 d	170.10 ± 2.315 c	124.36 ± 0.974 bc	217.92 ± 2.084 b

*Note:* Means followed by different letters in columns are significantly different at *p* < 0.05. Each group (free, bound or total) was analyzed and compared separately.

Abbreviation: db, dry basis.

## 4 Conclusions

In recent years, interest in functional foods and plant‐based proteins have been growing. In line with this interest, the present study aims to improve functional properties of traditional noodles (erişte) by supplementing with hull‐less barley and lentil flour in accordance with objectives of MEDWHEALTH project supported by PRIMA Foundation. Supplementation of erişte samples with 15%, 30% and 45% hull‐less barley or lentil flours resulted in increases in phenolic contents, antioxidant capacities and *β*‐glucan or protein contents besides decreases in GI values. All erişte samples supplemented with barley or lentil flours shifted from high‐GI foods to medium‐GI foods. The erişte samples supplemented with 45% hull‐less barley flour and Mix samples meet the requirements of the FDA health claim by providing the essential amount of *β*‐glucan per serving (0.75 g) divided over four meals. Moreover, the erişte samples enriched with 30% and 45% lentil flour can be labeled as “high in protein” while all other samples can be labeled as “source of protein” according to Regulation (EC) 1924/2006. It can be concluded that, hull‐less barley and lentil can be utilized as a source of *β*‐glucan and protein in erişte production. The results of this investigation can offer valuable insights that can support further research in the field of traditional foods with enhanced functional properties.

## Author Contributions

**Damla Arer:** data curation (equal), formal analysis (equal), investigation (equal), methodology (equal). **Oguz Acar:** conceptualization (equal), formal analysis (equal), investigation (equal), methodology (equal), supervision (equal), writing – original draft (equal). **Kubra Ozkan:** formal analysis (equal), methodology (equal), writing – original draft (equal). **Osman Sagdic:** writing – review and editing (equal). **Andrea Visioni:** resources (equal), writing – review and editing (equal). **Francesco Sestili:** conceptualization (equal), funding acquisition (equal), project administration (equal), writing – review and editing (equal). **Hamit Koksel:** conceptualization (equal), data curation (equal), funding acquisition (equal), methodology (equal), project administration (equal), supervision (equal), validation (equal), writing – original draft (equal).

## Ethics Statement

The authors have nothing to report.

## Conflicts of Interest

The authors declare no conflicts of interest.

## Data Availability

The data that support the findings of this study are available from the corresponding author upon reasonable request.
